# Antigenic Diversity, Transmission Mechanisms, and the Evolution of Pathogens

**DOI:** 10.1371/journal.pcbi.1000536

**Published:** 2009-10-16

**Authors:** Alexander Lange, Neil M. Ferguson

**Affiliations:** 1 MRC Centre for Outbreak Analysis and Modelling, Department of Infectious Disease Epidemiology, Imperial College London, London, United Kingdom; 2 Department of Mathematics and Statistics, McMaster University, Hamilton, Ontario, Canada; Emory University, United States of America

## Abstract

Pathogens have evolved diverse strategies to maximize their transmission fitness. Here we investigate these strategies for directly transmitted pathogens using mathematical models of disease pathogenesis and transmission, modeling fitness as a function of within- and between-host pathogen dynamics. The within-host model includes realistic constraints on pathogen replication via resource depletion and cross-immunity between pathogen strains. We find three distinct types of infection emerge as maxima in the fitness landscape, each characterized by particular within-host dynamics, host population contact network structure, and transmission mode. These three infection types are associated with distinct non-overlapping ranges of levels of antigenic diversity, and well-defined patterns of within-host dynamics and between-host transmissibility. Fitness, quantified by the basic reproduction number, also falls within distinct ranges for each infection type. Every type is optimal for certain contact structures over a range of contact rates. Sexually transmitted infections and childhood diseases are identified as exemplar types for low and high contact rates, respectively. This work generates a plausible mechanistic hypothesis for the observed tradeoff between pathogen transmissibility and antigenic diversity, and shows how different classes of pathogens arise evolutionarily as fitness optima for different contact network structures and host contact rates.

## Author Summary

Infectious diseases vary widely in how they affect those who get infected and how they are transmitted. As an example, the duration of a single infection can range from days to years, while transmission can occur via the respiratory route, water or sexual contact. Measles and HIV are contrasting examples—both are caused by RNA viruses, but one is a genetically diverse, lethal sexually transmitted infection (STI) while the other is a relatively mild respiratory childhood disease with low antigenic diversity. We investigate why the most transmissible respiratory diseases such as measles and rubella are antigenically static, meaning immunity is lifelong, while other diseases—such as influenza, or the sexually transmitted diseases—seem to trade transmissibility for the ability to generate multiple diverse strains so as to evade host immunity. We use mathematical models of disease progression and evolution within the infected host coupled with models of transmission between hosts to explore how transmission modes, host contact rates and network structure determine antigenic diversity, infectiousness and duration of infection. In doing so, we classify infections into three types—measles-like (high transmissibility, but antigenically static), flu-like (lower transmissibility, but more antigenically diverse), and STI-like (very antigenically diverse, long lived infection, but low overall transmissibility).

## Introduction

There are two major principles by which pathogens avoid their elimination: escaping the host immune response via antigenic variation or immune evasion, or transmission to a new immunologically naive host. Directly transmitted pathogens which cause chronic diseases, such as many sexually transmitted infections (STIs), tend to rely more on the former, while many acute infections, for instance measles, rely more on high transmissibility. Indeed pathogens such as measles show very little antigenic diversity, with immune responses being strongly cross-reactive between strains. There are then those pathogens which have intermediate levels of both immune escape and transmissibility — such as influenza, rhinovirus and RSV (here referred to as FLIs — flu-like infections).

The evolutionary success of directly transmitted pathogens can also be seen to depend on the nature, frequency and structure of contacts between hosts. Infections transmitted to a small number of hosts (per time unit and infected individual) via intense contact (e.g., via fluids) are usually caused by pathogens of high antigenic diversity and long duration of infection, while those transmitted via casual contact (e.g., via aerosol) with a large number of hosts may typically have lower diversity and much shorter durations of infection. While many of the evolutionary constraints are different [1],[2], vector-borne infections typically fall in the former of these two classes [3],[4]. The relationship between so-called infection and transmission modes with respect to substitution rates of RNA viruses has been investigated in [5].

It is straightforward to explain the long duration of infection and consequent antigenic diversity of sexually transmitted or blood-borne infections: the frequency of relevant contacts between hosts is low, meaning infection needs to be extended to ensure the reproduction number (the number of secondary cases per primary case [6]) exceeds one. However, many childhood diseases (ChDs) — at least those caused by RNA viruses — would also seem to have the genetic potential to prolong their survival within one host via by generating antigenic variants. The fact this is not observed is much harder to explain. At its root are the tradeoffs between maximizing between-host transmissibility and within-host duration of infection, and these are what we focus on exploring in this paper.

The molecular genetic basis of transmissibility is still poorly understood for most pathogens. However, all other things being equal, the level of pathogen shedding by a host (whatever route is relevant) must be positively correlated with infectiousness. A first-pass analysis might therefore postulate that overall transmissibility (as quantified by the basic reproduction number, 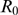) might be proportional to the total number of pathogen copies produced during an infection — the cumulative pathogen load. Past work using a simple model of the interaction between a replicating pathogens and adaptive host immune responses examine what rate of antigenic diversification within the host would maximize cumulative pathogen load [7]. This showed that the combination of resource-induced (whether nutrients or target cells) limits on peak pathogen replication rates and an ever more competent immune response mean that the optimal strategy is not to diversify as rapidly as possible, but instead to adopt an intermediate rate of diversification. In addition, there are further tradeoffs associated with high mutation rates — the ultimate being the error catastrophe associated with error rates in genome replication which exceed those seen in RNA viruses [8]–[11].

**Figure 1:**
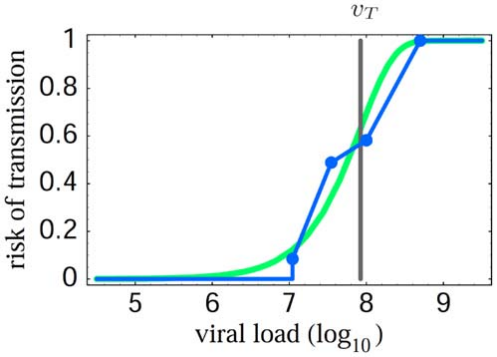
Risk of HIV-1 transmission as a function of viral load, using data from[12]. The maximal risk, which corresponds to 11.8 per 100 person-years, is normalized to 1, and the viral load in 10 liters of plasma plotted. Data points (blue polygon) are compared with the least-squares best fit of the infectiousness model given in the text (green); cf. (7). The viral load 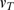, fitted by the data, indicates the order of magnitude needed for a substantial probability of transmission — this is the load of pathogen referred to as infectiousness threshold (gray).

However, the assumption that transmission fitness (as quantified by 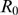) is linearly proportion to total pathogen load is clearly naïve. The instantaneous hazard of infection for a susceptible host in contact with an infected host at a point in time may indeed be linearly related to pathogen load at that time, but going from this assumption to a calculation of the overall reproduction number is far more complex than simply calculating the area under the pathogen load curve. Integrating a hazard over the finite time of contact gives an exponential dependence between the probability of infection 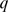 and pathogen load 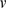, i.e., 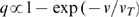. Such an expression fits experimental data [12] on the relationship between HIV viral load and transmission rates well (cf. Fig. 1). This means the parameter 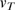 represents a pathogen load threshold below which the probability of infection declines rapidly, and above which it rapidly saturates to some maximal value. Hence 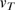 can be thought of as the characteristic pathogen load required for transmission — though it is not a true minimum infectious dose — there is a finite probability of infection for 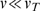 , but that probability decays exponentially fast with reducing 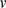.

A key insight (and assumption) of the work presented here is that while we might expect pathogens to be able to evolve to reduce (or increase) 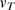, there are fundamental physical constraints imposed by transmission routes on the minimum value of 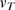 attainable. An STI might have a minimum value of 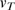 approaching a single pathogen particle (e.g. virion) but, for respiratory infections, the much lower proportion of all pathogen particles emitted from a host, which have any chance of contacting epithelial tissues of a susceptible host (even conditioning on a susceptible host being in the near vicinity of the infected individual), necessarily means that 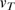 must be orders of magnitude larger for such pathogens.

We will show that there is a critical value of 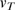 above and below which two different sets of pathogen types are evolutionarily favored (in terms of having maximal 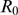). Within each set, the particular type which has maximal 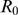 will be seen to depend on the local structure of the contact network between hosts.

Our approach is to construct a model of within-host pathogen dynamics which incorporates adaptive host immunity and antigenic diversification. The key output from this model is how pathogen load varies through time during an infection. We then calculate the basic reproduction number, 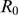, for that infection assuming a particular local contact network structure and frequency of contacts.

The within-host model developed here is an extension of a model studied earlier by one of us [7]. Our work builds on a range of past work examining the tradeoffs between within-host replication and persistence, antigenic variation and between-host transmission success, initiated by [13], and followed by [14],[15], which first include immune response and explore cross-immunity. More recent studies, to mention a few, investigate pathogen evolution under limited resources [16], include virulence [17], consider the immunological response in more detail [18], examine the impact of between-host contact structure on pathogen evolution [19],[20], and explore host-pathogen co-evolution [21],[22].

We use 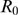 as our fitness measure for determining evolutionarily optimal phenotypic strategies. We do not explicitly model competition between pathogen strains with different phenotypes co-circulating in a host population, since for infinite populations, 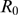 has been shown to be the fitness measure which determines the outcome of such competition [23]. This holds even when comparing strains with different rates of antigenic diversification — if the strain with lower 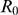 induces no long-lived immunity in the host (giving SIS dynamics) and the higher 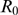 strain induces life-long immunity, (giving SIR dynamics) the higher 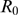 strain will still always (eventually) outcompete the lower 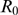 strain. There are limitations to the use of 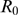 as a fitness measure (further considered in the Discussion) — for instance, in situations where strains interact asymmetrically via cross-immunity, or when populations are small and stochastic extinction is significant. In addition, while we take account of local (egocentric) network structure in defining 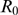 in our analysis, large-scale network structure might also affect the determinants of evolutionary fitness. However, we feel these limitations are outweighed for an initial analysis by the analytical and computational tractability afforded by use of a relatively simple transmission measure, and the consequent ability not to rely on unintuitive large-scale simulations.

We do not explicitly consider how a pathogen could evolve its biological characteristics to maximize transmission fitness (i.e. the evolutionary trajectory a pathogen would take through parameter space). There are undoubtedly many constraints on the possible paths which pathogens can take [24], however, and exploring how these affect, for instance, pathogen adaptation to a new host species, will be an important topic for future work.

## Results

### Within-host dynamics

The multi-strain model used extends past work [7] by adding cross-immunity between strains (see Methods for details). The infection within one host starts with a single strain, with further strains arising through random mutation. All strains compete for resources (e.g. target cells) to replicate. Immune responses to strains are assumed to be predominantly strain-specific, albeit with a degree of cross-immunity, the strength of which decays with the genetic distance between strains. Pathogen replication depletes resource, and independently from immunity, limits to pathogen growth are set by the replenishment rate of resource. This quantity only determines the short-term dynamics of the model whereas immunity is also responsible for the long-term behavior.

The dynamics of the model is characterized by an initial period of exponential growth of the pathogen load, which eventually slows due to immune responses and resource limitations. One observes a latency period and an initial peak. Pathogen load then declines exponentially. If the trough load of a pathogen strain drops below a threshold level we assume the pathogen is eliminated from the host (to avoid persistence at unrealistically low, fractional, loads). However if a novel strain emerges before the seed strain goes extinct, pathogen load can recover, so long as there is sufficient resource available and cross-immunity is not too strong — leading to a second, albeit lower peak in pathogen load. Further peaks in pathogen load can occur via the same mechanism. The rate at which new strains arise is the most important determinant of the number of pathogen load peaks seen and thus the overall duration of infection. Less intuitively, this rate also determines the size of the initial peak (discussed below).

**Figure 2:**
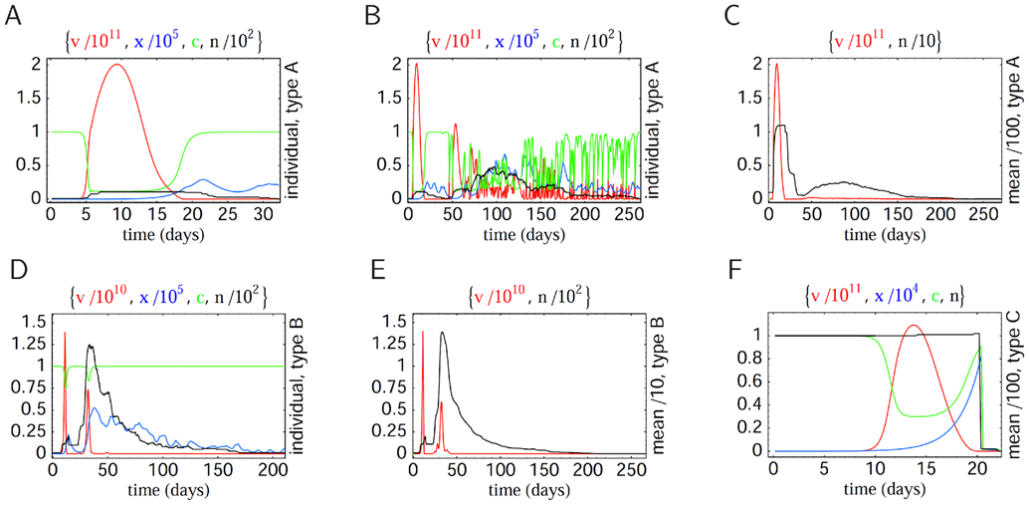
Within-host model dynamics. Graphs show pathogen load [red], specific immunity [blue], resource [green], number of strains [black] and corresponding mean values plotted over time — for individual hosts in (A,B,D) and average hosts in (C,E,F), respectively. (A) and (B) show two different model realizations for the same parameters of antigenic mutation proportion 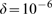 and replication rate 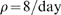, defining type A infections, cf. the Methods section and Fig. 3. One observes extremely different durations of infection — reaching from a few days up to one year. (C) shows the corresponding average behavior over 100 realizations, characterized by low pathogen loads at large times. Determined by mean load values, this infection type corresponds to intermediate and low mean durations of infection — much shorter than the approached maximum of one year. This is also reflected by the mean strain number, which reaches a maximum of 10 at the initial load peak, drops down to almost zero and rises again slowly to values of about 1 for a few months. (D) and (E) show the pathogen dynamics specific to type B infections with 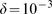 and 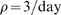, for individual and average hosts, respectively. The mean values of load and strain number coincide with the individual values, which confirms long durations and high strain numbers as characteristic trait of this infection type. (F) illustrates type C infections through average curves (over 100 runs) at 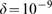 and 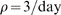; mean and individual values coincide almost identically as the average strain number is close to 1.

Since mutation is modeled stochastically, we average over multiple realizations (e.g. Fig. 2A,B) of the model to calculate an average pathogen load distribution over time (Fig. 2C). The average distribution consists of a first latency period, a large initial peak, a second latency period and possibly an irregular oscillating part of low pathogen load. The point at which the viral load vanishes determines the duration of infection.

We systematically calculate average pathogen load curves from the within-host model for wide ranges of two biological parameters: the antigenic mutation rate 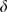 (i.e., the rate of mutations which lead to antigenically novel strains) and the pathogen replication rate 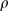. These two parameters span what we call *pathogen parameter space*, in which evolutionarily favored pathogens are represented by points that are associated with maximal fitness values.

**Figure 3:**
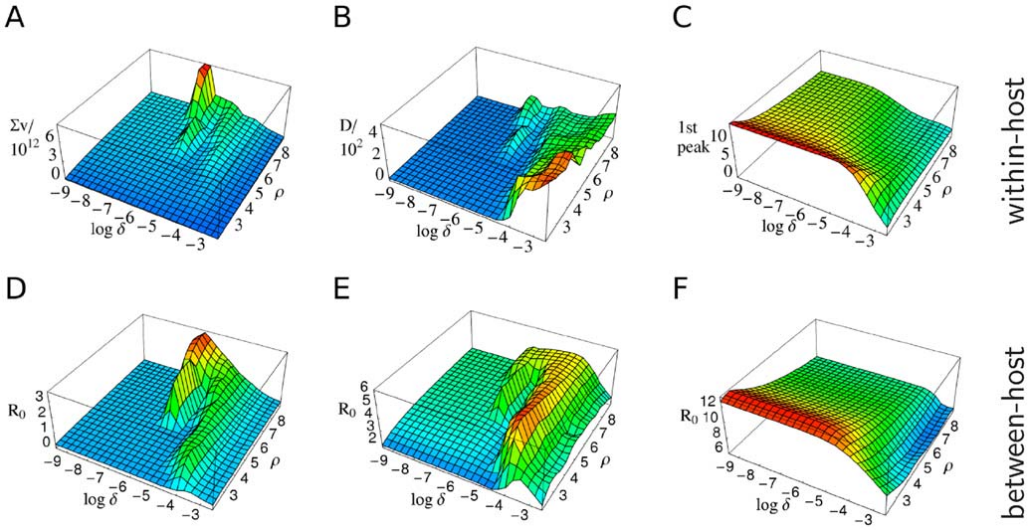
Qualitative relationship between between-host 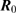 and within-host dynamics as a function of parameters governing within-host antigenic diversity 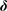 and replication rate 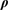. (A) the cumulative pathogen load 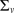; (B) the duration of infection 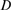; (C) a combination of the latter two at the initial load-peak (relevant for transmission, i.e., 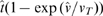, quantified at the 1st peak 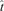 of the pathogen load 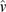, cf. (6)). The reproduction number 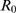 is calculated for a transmission network with (D) low [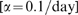], (E) intermediate [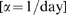], (F) high contact rates [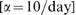], where network parameters and infectiousness and are utilized that allow for ChDs (i.e., 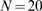, 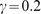, 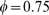, 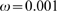, and 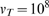). Within-host parameter values are set to default values as given in the Methods section. The shapes of the surfaces and the locations of the maximums are similar for the upper and lower row, i.e., for (A) and (D), (B) and (E), (C) and (F). The three distinct pairs of locations of the maxima correspond to our infection-type classification — representing FLIs, STIs, ChDs, respectively.

From the discussion in the introduction, we can immediately identify the cumulative pathogen load and duration of infection as epidemiologically relevant quantities. Fig. 3A,B show these as a function of the parameters 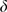 and 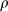. In addition, Fig. 3C shows a quantity — interpolating between the two former — evaluated only for the initial period of the infection (utilizing the expression relevant for transmission, i.e., 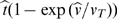, quantified at the initial peak 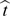 of the pathogen load 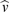). We will see below that all the surfaces shown in Fig. 3A–C crudely represent fitness surfaces associated with three distinct pathogen types. The plots in Fig. 2 show the corresponding within-host dynamics for the different pathogen types.

The within-host dynamics generate a tradeoff between initial peak pathogen load and antigenic diversity: high initial peak load corresponds to low diversity and vice-versa (see Methods for more details). This tradeoff has implications for transmission, giving an enhanced spread of pathogens of low antigenic diversity during the initial peak of pathogen load. This effect explains the emergence of (ChD-like) infections with short durations of infection within our model framework (Fig. 3C vs. 3F). Long durations of infections (Fig. 3B) are also obtained, as expected, for pathogens with greater antigenic variation.

### The between-host model

To calculate the reproduction number (i.e., the pathogen fitness), we model a dynamic contact network in the neighborhood of one initially infected host. The profiles of pathogen load over time obtained from the within-host model then determine the infectiousness of the infected host to its neighbors. (We utilize the mean-load profiles averaged over individual hosts.) Epidemiological dynamics are determined by 4 parameters. Two of these relate to properties of the transmission route: the infectiousness parameter 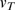 and the contact rate between hosts 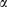. Together these define a two-dimensional parameter space we term *transmission space*. The other two define properties of the contact network between hosts: the replacement rate of neighbors 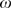 and the cliquishness/clustering of the network 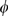 (i.e., the proportion of pairs of contacts of a host who are also contacts of each other). These two parameters define what we term *contact space*.

We build a model (cf. Methods) incorporating these 4 parameters (plus implicitly the within-host pathogen space parameters) to calculate the number of first generation infections from an infected individual in an entirely susceptible population.

Varying the 4 parameters of transmission and contact space, we obtain three different classes of fitness landscapes over pathogen space — as represented by Fig. 3D–F. The maxima of each landscape differ with respect to their antigenic mutation rate (and hence the resulting level of antigenic diversity) and within-host pathogen replication rate. By changing the contact rate and keeping the other transmission as well as the contact space parameters fixed, one can shift between these classes. In general (as shown further below), low, intermediate, and high contact rates induce moderate, high, and low antigenic diversity, respectively, as evolutionarily favored outcomes (represented by the locations of the fitness maximum in Fig. 3D–F).

### Infection types

There are clear similarities between the three classes of fitness landscapes (Fig. 3D–F) and the different within-host infection characteristics plotted in Fig. 3A–C. Low contact rates induce landscapes that resemble the cumulative pathogen load, intermediate contact rates give landscapes resembling the the duration of infection surface, and high contact rates map onto the surface of Fig. 3C which characterizes the relative importance of the initial peak in the pathogen load profile. We classify the optima of these 3 classes of fitness landscape infection types, labeling them A, B, and C, respectively.

Varying the infectiousness parameter 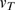 can also move the fitness landscape between these types — as 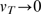 (the STI limit; i.e., 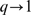, 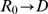), the fitness landscape becomes more similar to the duration of infection surface (Fig 3B), while for 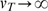 (the FLIs limit; i.e., 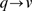, 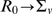), it becomes more similar to the cumulative pathogen load surface (Fig. 3A); cf. (7) and (6). It is important to note that both of these limits involve substantial antigenic diversity — where transmission fitness is dominated by cumulative pathogen load (infection type A), while moderate antigenic diversity is seen, and when infection duration dominates fitness (infection type B), high antigenic diversity is selected for. Neither maps on to the special case of infection type C (Fig. 3F) in which optimal transmission fitness is achieved by a set of parameters giving very low antigenic diversity (in essence a single strain). For low antigenic diversity to be optimal, it is necessary for fitness to be dominated by the peak pathogen load achieved during primary infection (i.e., the first peak of pathogen load).

**Figure 4:**
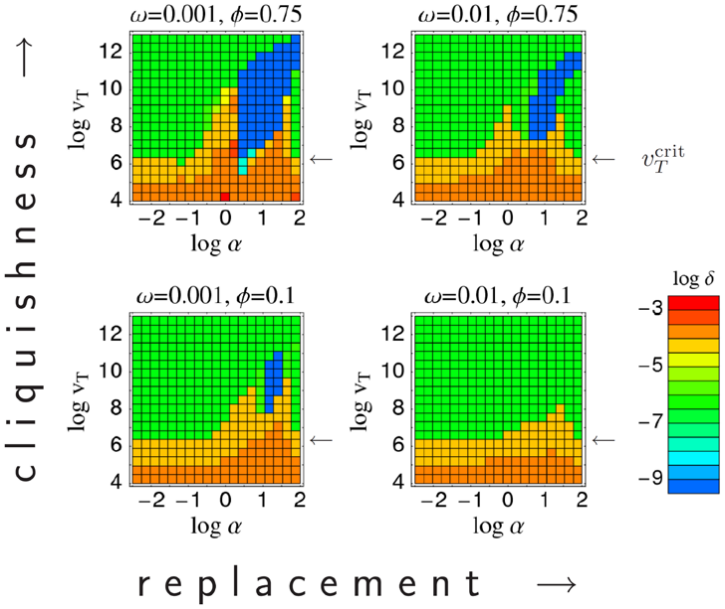
Evolutionarily optimal antigenic diversity as a function of epidemiological contact rate 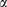 and the infectiousness 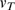. Different plots show results for different choices of between-host contact network, as defined by the replacement rate of network neighbors 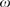 [horizontal] and the cliquishness 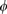 [vertical]. For each set of parameters, pathogen space parameters are tuned to give optimal transmission fitness (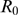). The color indicates the degree of antigenic diversity (represented by the value of 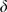 giving maximal 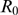) seen for the evolutionarily optimal point in pathogen space [blue = low diversity, red = high diversity]. Blue represents single strain ChD-like type C pathogens, which are not present for low network cliquishness and high replacement rates (bottom right quadrant). Green represents intermediate antigenic diversity type A pathogens, while orange and red represent high antigenic diversity type B pathogens — the arrows indicate the critical infectiousness threshold 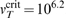. Maximum transmission probability per contact assumed to be 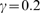, with network neighborhood size of 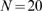 (typical of ChDs).

Varying the transmission and contact space parameters more systematically, one can map out the regions of parameter space for which particular infection types are optimal (Fig. 4). This shows how the emergence of pathogens of different types depends on the properties of the between-host contact network. Pathogens with low antigenic diversity (and thus short infectious periods) are favored by high network cliquishness (i.e., when an individual's contacts are contacts of each other — as is the case for household and school contacts), and the rate of turnover of network neighbors is low (again the case for household and school contacts).

**Figure 5:**
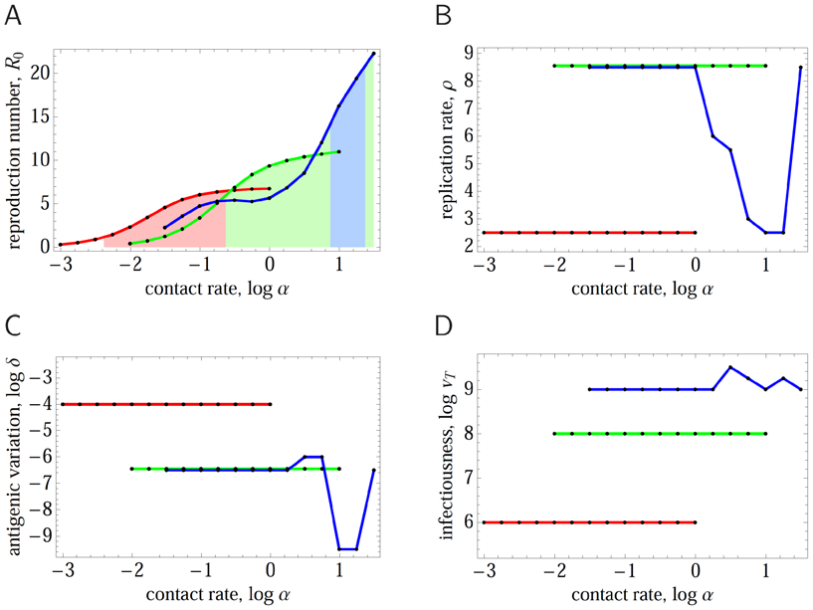
Evolutionarily optimal pathogen parameters as a function of the epidemiological contact rate, 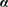 (in units of 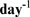). For each value of 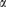, the infectiousness threshold 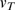, within-host replication 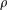, and antigenic diversity 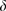, are tuned to maximize the reproduction number 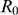. (A) 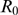 [colored areas indicate the infection type according to the corresponding 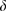 value, and 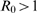]; (B) 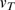 — subject to minimum bound 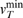; (C) antigenic variation rate 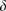; (D) replication rate 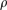. Three sets of results [colored curves] are shown, for network parameters typical of STIs [red], FLIs [green], and ChDs [blue]. The following parameters were used: 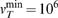, 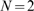, 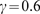, 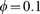, for STIs; 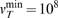, 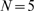, 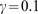, 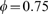, for FLIs; 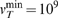, 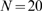, 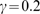, 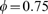, for ChDs; and 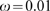 in all three cases. (We only examine the corresponding biologically realistic regimes of 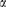, discretized as indicated by dots.) The results demonstrate how the infections of our type-classification outcompete each other for different host-contact rates.

So far we have assumed only the pathogen space parameters (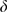 and 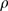) can change during pathogen evolution. Now we examine making the infectiousness threshold 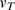 a parameter which can evolve under selection — albeit with constraints on its lower bound set by the transmission route of the pathogen concerned. Fig. 5 shows the results as a function of contact rate 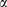 for two different choices of contact space parameters and lower bounds on the infectiousness threshold parameter, suitable for a respiratory pathogen and an STI respectively. Reproduction numbers (Fig. 5B) lie in the expected range, and the three regimes of antigenic diversity corresponding to the types A/B/C) can be found in the evolutionarily optimal values of 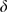 (Fig. 5A,C). Note that only type A and type C diversity is seen for the respiratory pathogen parameter choices, while only type B is seen for the STI parameter set. Indeed for the STI parameter set, the evolutionary stable state is independent of the contact rate, and is determined by 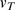 evolving to its minimum value.

As expected, the evolutionary optimal value of the infectiousness parameter (Fig. 5B) is always close to the minimal attainable value, except in the type C pathogen regime (where cliquishness is necessary; cf. Fig. 4). The reason for the deviation from the minimum value lies in a reduced local network saturation, which is characteristic for type C: concentrating infectiousness over the shortest possible time period (and consequently lengthening the latent period) shortens the overlap between generations of infections, and this reduces the chance that the secondary cases of an index case infect remaining susceptible contacts of the index (before the index can infect them). The effect (which yields an enlarged susceptible number 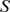 in (6)) is minor, however — the difference in 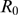 between the optimal value of 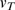 and the minimum bound set for a pathogen type is typically very small.

The evolutionarily optimal replication rate 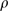 is always low for STI-like contact parameters (giving type B pathogens), reflecting the need for long-lived infections, but shows greater variability for respiratory pathogen parameter regimes (Fig. 5D) — being high in the type A regime, but low for type C. The latter result reflects a tradeoff between height of the initial peak in pathogen load and length of the latent period — longer latency, as explained above, can increase the number of direct infections caused by an index case by reducing the overlap between generations of infection. Only higher (minimal) infectiousness values 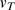 — realistic for ChDs utilizing the respiratory transmission route — increase the optimal replication rate for type C infections (cf. Text S1, Sect. B.2). Note that these results are consistent with a recently formulated hypothesis on tradeoffs between reproductive rate and antigenic mutability [25], proposing a reciprocal relationship between these two (pathogen space) parameters in real-world infections.

**Figure 6:**
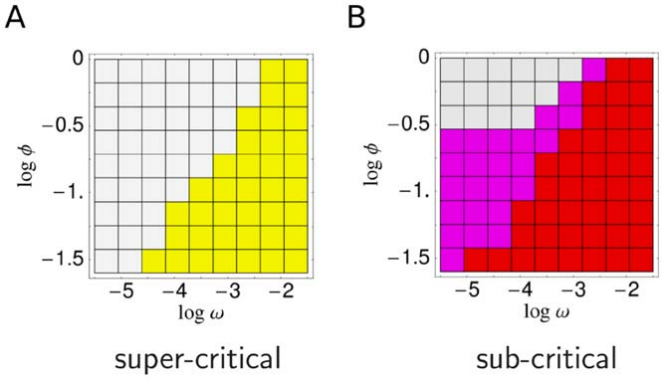
Evolutionarily favored infection types as a function of contact network parameters. (A) results for a lower bound on 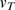 of 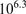 [super-critical, 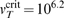]; (B) results for a lower bound on 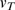 of 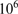 [sub-critical]. The colors represent which combinations of infection types can exist for particular values of contact space parameters: ABC [white], AB [yellow], BC [purple], B [red]. Exactly which infection type is evolutionarily optimal is then determined by the contact rate 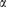 (cf. Fig. 5A).

Re-examining Fig. 4, it is clear that type A infections (green areas) only exist when the infectiousness parameter 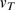 exceeds some minimum value (indicated on the graphs in Fig. 4 with an arrow). In the absence of constraints, selection for maximal transmissibility will clearly cause 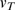 to evolve towards 0. Hence the effect of constraints on imposing a lower bound on 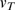 has a critical effect on what range of pathogen types are expected. We define the value of the lower bound on infectiousness below which infection type A is no longer found the *critical infectiousness threshold*. Evolutionary dynamics show a phase transition at this point, as can be seen in Fig. 6 which maps the areas of contact parameter space for which different infection types are seen for choices of the lower bound on 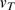 just above and below the critical point 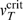.

As discussed already, the transmission route is likely to be the most important determinant of the lower bound on 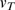, with STIs and other non-airborne pathogens, including those requiring a vector, being likely to achieve a much lower value of 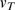 than respiratory pathogens (as assumed in Fig. 5). This is clear if one views 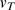 as quantifying how much shed pathogen is typically wasted to achieve a single infectious contact. We therefore speculate that the critical infectiousness threshold may have a significant biological effect, with STIs — and also vector-borne infections — being within the sub-critical domain (Fig. 6B), and with ChDs and FLIs — not necessarily relying on a respiratory transmission route — being super-critical (Fig. 6A). Within the super-critical regime, the presence of low-diversity ChD-like type C infections depends less on the precise value of the critical infectiousness threshold and more on the contact rate and contact parameters. Infections of type C occur in contact networks with high cliquishness and low replacement rates — but not in the opposite case (cf. presence of blue areas in Figs. 4 and 5A). Vector-borne infections (representing contact networks of large neighborhood sizes 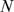 or high replacement rates 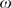, and cliquishness 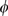 not playing a role) are thus excluded to be type C. At first sight they seem to be type A, because of large reproduction numbers. Large 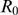, however, can also be the result of large neighborhood sizes or high replacement rates — immediate from (6) and (8). The quantity being important in this context is the lower bound on possible infectiousness values, which is small (i.e., sub-critical, 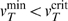) — this identifies vector-borne infections as type B.

## Discussion

The work in this paper was motivated by a desire to understand why the most transmissible human pathogens — archetypal childhood diseases such as measles and rubella — show remarkably little antigenic variation, while less transmissible diseases — such as influenza (and many other respiratory viruses) and sexually transmitted diseases show substantial diversity. Addressing this question requires consideration of how evolvable parameters governing the natural history of infection within a host affect the transmission characteristics of a pathogen in the host population.

We developed a relatively simple multi-strain model of the within-host dynamics of infection. Pathogen particle consume resource to replicate, and their replication is inhibited by a dynamically modeled immune response with two components: strain-specific immunity, and cross-immunity. Cross-immunity was assumed to be the key fitness cost of antigenic diversity within the host; the benefit is a much enhanced duration of infection (and thus transmission). Pathogens which have a low rate of generating new antigenic variants are cleared from the host much faster than those with a high rate of antigenic diversification, but also maximize the initial peak level of parasite load reached prior to clearance (cf. Methods).

The second evolvable within-host parameter we considered was the within-host pathogen replication rate. Given the resource-dependent model of replication assumed, this has a more limited effect than in some models, but can set the timescale for pathogen load to initially peak and thus determine the effective latent period of the disease.

At the between-host level, we assume a simple relationship between pathogen load and infectiousness which has been shown to be appropriate to model HIV transmissibility [12], and incorporates the concept of a soft threshold level of pathogen load needed for a substantial level of transmissibility, 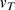. As argued above, this parameter is perhaps best viewed as the amount of excreted pathogen which is wasted to achieve an infectious contact. For a perfect pathogen, the value could correspond to a single pathogen particle, but in reality the physics of transmission will typically mean 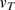 is much higher. We have considered 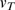 to be an evolvable parameter, but introduced the concept of minimum possible value of 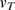 which is transmission route specific — being intrinsically much higher for respiratory pathogens (where transmission occurs via virus filling a three-dimensional volume around the infected individual), and potentially much lower for sexually transmitted diseases where transmission occurs over a two-dimensional contact surface.

The final element we incorporate into the framework developed is contact between hosts, assumed to occur at some rate 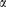, within a contact network of hosts with a certain mean neighborhood size 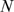 and cliquishness 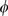. We derive a simple model to calculate the reproduction number of a single infected host in this network allowing for local saturation effects in the network caused by clustering. It is the network-specific reproduction number we have used as our overall measure of pathogen fitness, and examine what within- and between-host pathogen characteristics maximize fitness for different types of transmission route and host contact network.

Putting these elements together, we found that optimizing reproductive fitness in this way leads to well-defined infection types A, B, C, as contact rates (and reproductive numbers) increase (cf. Fig. 5). Type A and B both represent infections with low 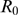, with A being influenza-like and B mapping more to sexually transmitted diseases. When contact rates are very low, only one of these two types is evolutionary stable, with the stable type being determined by the assumed minimum infectiousness threshold. The latter serves as an order parameter and determines the mode of transmission. Consistently, type A corresponds to a high minimum infectiousness threshold whereas type B results from a low minimum threshold. The change of the transmission mode as a function of transmission threshold is phase transition-like.

Infection type C represents childhood diseases with the highest values of 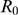. This regime is not possible for small network neighborhood sizes or low values of cliquishness (i.e. random networks). It relies on the existence of large, persistent and highly clustered contact neighborhoods. In this context, maximizing the number of secondary infections (and thus overall fitness) requires a pathogen strain able to (a) infect as many of the index host's contacts as possible in as short a possible time, and (b) minimize the extent to which generations of infections overlap. The latter constraint is a result of the network clustering — if secondary cases become infectious while the index case is still infectious, they may deplete susceptible from the contact neighborhood before the index case has the chance to infect them. A latent period of the same or longer duration as the infectious period results in more discrete generations and maximizes the reproduction number of the index case. The need for a long latent period results in the evolutionary optimal value of the within-host replication rate 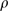, being relatively low for type C pathogens.

The limited antigenic diversity and short infectious periods of type C pathogens are determined by the higher infectiousness threshold and the consequent need to maximize the peak pathogen load attained early in infection. When contact rates are high, the increase in duration of infection resulting from higher rates of antigenic diversity is insufficient to compensate for the reduction in peak pathogen load (and therefore infectiousness) caused by cross-immunity being generated against multiple pathogen strains simultaneously. A single strain pathogen generating a single immune response is able to generate a larger primary infection peak — though at the cost of being unable to sustain infection further.

It is encouraging to see that the classification of infection types our model predicts closely corresponds to many of the pathogen regimes identified in other work [24]. However, our focus has been slightly different from that work, which focused more on the effect of different intensities of cross-immunity on between host phylodynamics. In contrast, we have focused more on examining how differences in transmission routes and contact rates (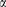) determine pathogen characteristics — though the influence of different levels of cross-immunity could be explored in future work.

Furthermore, it is interesting to note that in the context of our model only the concept of a minimal infectiousness threshold — introduced to characterize transmission modes — is necessary to explain the findings of [25] on tradeoffs between reproductive rate and antigenic mutability. Reference to the host's age is not needed here.

The key limitation of our analysis is our highly simplified treatment of between-host transmission — namely using a network-corrected reproduction number as our measure of strain fitness. Doing so assumes evolutionary competition occurring in infinite (non-evolving) host populations in infinite timescales. It would clearly be substantially more realistic to explicitly simulate the transmission process in a large host population. The computational challenges are considerable — while large-scale simulations of influenza A evolution and transmission have been undertaken [7],[26],[27], these have not included within-host dynamics, and have simulated evolution for decades rather than millennia. Other work [20],[28] has simulated the evolution of pathogen strains on a contact network for longer time periods, but only in very small (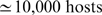) populations, and without modeling within-host dynamics.

However, continuing advances in computing performance mean that it may now be feasible to explicit model multiple strains evolving within hosts and being transmitted independently in a large population. Such an approach would allow exploration of the relationship between antigenic diversity (and cross-immunity) within single hosts and strain dynamics at a population level. Perhaps even more importantly, it would allow extinction processes to be properly captured, while our current approach implicitly assumes fixation probabilities to be 1 even when fitness differences are marginal. Proper representation of finite population sizes and extinction will also allow the evolutionary emergence of childhood diseases (such as measles) as a function of early urbanization to be modeled.

A second limitation is that we only consider a single, highly simplified within-host model. Future work to test the sensitivity of our results to the choice of within-host model would be valuable (cf. Text S1, Sect. A, which investigates an extension of the model here). That said, we would argue that the key qualitative feature of our within-host model driving the evolutionary results is the tradeoff — mediated by cross-immunity — between the maximum value of parasite load attained in initial infection and the degree of antigenic diversity (and thus duration of infection).

Also a conceptual simplification must be pointed out here: our model assumes that mutations, controlled by 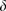, directly affect antigenicity. For real-world pathogens, however, the link between genetic and antigenic change is less clear. Measles, for example, has a mutation rate typical of RNA viruses [29], but its antigenic diversity is low. Instead of mutation rate controlling antigenic variability, a pathogen may evolve phenotypic robustness to genetic change.

Further, we have not attempted to capture specialized strategies pathogens have adopted for persistence within infected hosts, such as use of refuges from immune responses (HSV) or hijacking the immune system (HIV) — the model only reflects tradeoffs which may have contributed to pathogens adopting the range of persistence strategies seen in nature. An interesting addition to future work would also be the incorporation of pathogen virulence [30], which imposes an additional evolutionary constraint on within-host replication rates.

A last area which is a clear priority for future research is the relationship between within-host parasite load and infectiousness. We have assumed a relationship which has some support in data (Fig. 1), and indeed the HIV system is perhaps the best explored in terms of the possible evolutionary tradeoffs inherent in maximizing transmissibility [31]. Unfortunately, little comparable data is available for other (especially respiratory) pathogens.

## Methods

### Within-host model

The within-host dynamics are simulated by the following system of ordinary differential equations (see [7] for more details where this system is introduced without cross-reactive immunity):
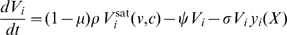
(1)

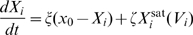
(2)

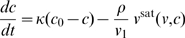
(3)representing (1) the load of pathogen strain 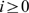, (2) the amount of the adaptive immune response specific to strain 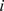, and (3) the level of resource which all strains need to replicate; the number of equations, 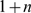, corresponds to the number of strains present, where 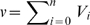 denotes the total pathogen load. For viral infections, for example, the load 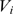 is assumed to represents the number of virions of strain 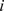, the immunity variable 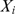 somehow the amount of specific antigen (produced by B cells), and the resource 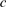 target cells (e.g., epithelial cells for flu or T cells for HIV) of maximal number 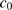.

Saturation effects, modifying linear dependency on 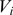 and 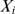, are modeled with the Hill function 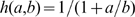. The resource limitations act via 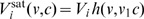, where, for large loads (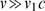), growth is limited by the maximal pathogen capacity related with the resource, 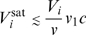; for small loads, the load is independent of the resource, 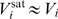. The adaptive immune response is given by the growth term 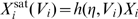, which increases in response to antigen quickly and reaches values 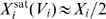 at 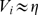. For larger pathogen loads, growth stops slowly, limited by 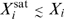 when 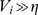. The parameter 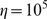 represents the critical load above which immunity saturates. Its value is chosen above the number of pathogen units 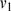 released after one replication cycle per resource unit (see below).

Guided by values for RNA viruses, random mutations are assumed to occur with probability 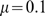 per pathogen replication, which happens at rate 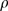. Only a proportion 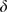 of mutations generate new antigenic variants. We assume that all mutations not leading to new antigenic variants are deleterious. The emergence of new antigenic variants is modeled stochastically, where a Poisson distribution with expectation 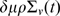 determines the number of mutant strains 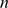 at time 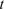, with 
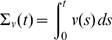
 denoting the cumulative load. While back mutations are neglected in the equations above, they are taken account of in the numerical calculations.

New antigenic variants 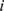 generated at time 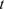 induce a specific immune response, 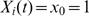. This grows so long as 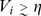, and declines for 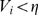 downwards, but never goes below 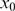. These characteristics are determined by the structure of (2) and the parameter choice 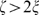, where 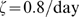 and 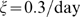 define the base rates at which immunity is produced and declines, respectively.

We assume 5 loci with 3 alleles at each. (These numbers are small but sufficient for our analyses, cf. Text S1, Sect. C.) The distance between strains, 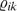, is defined as the number of loci at which strains 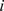 and 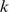 differ. The immune-related clearance rate of strain 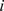 is given by 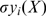, where 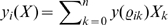 and 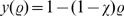 for 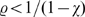 and 0 otherwise. Here 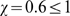 is the degree of cross-immunity, and 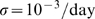 is the parameter governing homologous clearance rates.

Independent of immunity, pathogen is cleared at a rate 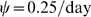 (chosen smaller than 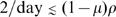; cf. (5) below). Pathogen growth is limited by resource, where 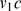 defines the saturation point. As pathogen grows at rate 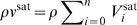, resource is consequently depleted at rate 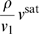. Resource is replenished at rate 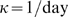, and its total is modeled to never exceed 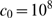 (chosen to represent a realistic number of target cells and thus give realistic pathogen loads; cf. Fig. 1 and the examples above).

The differential equations are solved using a Runge-Kutta algorithm with the initial values 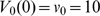, 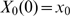 and 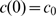, starting with 1 strain. New antigenic variants are generated potentially after each time step, each with initial pathogen load 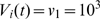 (corresponding to 1 pathogen unit infecting 1 resource unit) and specific immunity 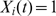, if generated stochastically at 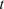. The infection ends once pathogen load drops below the value 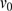 (which is assumed to be the elimination threshold), or after 2 years (the latter cutoff being chosen for computational simplicity).

The parameter values (essentially 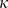, 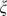 and 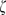) and the regions of pathogen space (given by 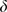 and 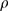) have been chosen to produce load curves (with significant resource depletion at the load peak, i.e., 
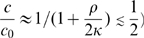
) that resemble measles characteristics (with latency periods of up to 10 days and significant pathogen loads for similar periods; cf. Fig. 2F) for small antigenic variation and small/intermediate reproduction rates. The duration of infection is adjusted by the strength of immunity (i.e., 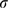), with the value used here selected to give infections of over 1 year duration for maximal antigenic variation.

This model is minimally complex, incorporating only the features essential to explain the tradeoff between transmissibility and antigenic diversity. A more realistic model is examined in Text S1, Sect. A. However, the key diversity-transmissibility tradeoff arises as a simple consequence of within-host cross-reactive immune responses raised to individual new strains and competition between strains for a common resource for replication, and is relatively independent of the model-specific form of implementation of these mechanisms.

The essential within-host dynamics of our combined within/between-host model is given by Eq. (1), which links pathogen replication to two inhibitors — host immunity and resource limitation. This equation quantifies the tradeoff for increasing antigenic diversity (the pathogen's survival strategy within the host) — namely the smaller initial pathogen load peak seen in Fig. 3C (and Fig. S1-2C in Text S1, Sect. B.1). The specific realizations for the acquisition of immunity and the replenishment of resource (modeled by Eqs. (2) and (3), respectively) are less important.

Let us consider the pathogen load dynamics soon after infection with one initial strain. Our numerical simulations have shown that the initial strain 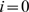 is much more prevalent (by orders of magnitude) than mutant strains produced up to the first peak, 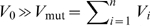. This observation clarifies that resource limitation (as one inhibitor of pathogen growth) cannot explain the tradeoff discussed here — being of low prevalence, mutant strains are unlikely to deplete resource to an extent which results in significantly lower loads, and in any case all strains have the same intrinsic replication rate and use the same resource. But the specific immune response to mutant strains, provided it is partially cross-reactive, is able to reduce both the load of the initial strain *and* other strains, and can thus lower the total pathogen load. This result is largely independent of model implementation and only depends on the strain-specific immune response being generated at relatively low strain-specific pathogen loads, and being sufficiently cross-reactive to slow overall growth of pathogen load.

This verbal argument can be formalized. For simplicity we assume the load of the initial strain is a good approximation of the total pathogen load at the initial peak, 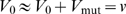. By applying 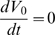 as a condition for the initial peak, Eqn. (1) (with 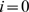) then yields a relation for the initial peak load,
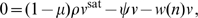
(4)where 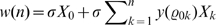 defines the immune response with respect to the strain number. Provided cross-reactive immunity is implemented (i.e., 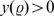 for some 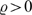, so that 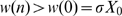), the function 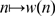 is strictly increasing (independently of how cross-immunity is defined via the strain-distance weight function 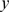 and the parameter 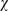). This is based on the fact that, together with each newly generated strain 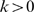, immunity 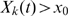 is produced in a standardized way for the time period up to the initial peak when load is increasing and above a critical value, 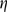. This is the case in any setting where mutant strains have the same intrinsic replication kinetics as the initial strain. In our model, immune production happens at rates above 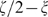 (and below 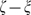) as long as 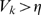, independently of the concrete acquisition rule in (2); see the modifications (Eqs. (S1-1,2)) and the corresponding result (Fig. S1-2C) in Text S1 for a more realistic but also more complicated mechanism.

As a consequence of resource limitation (i.e., the reduced growth 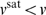), (4) yields
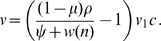
(5)Due to the monotony of 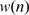, the function 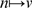 given by (5) is strictly decreasing. That means that the magnitude of the initial peak 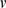 is inversely related to the number of (mutant) strains 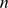 present. The result is independent of the specific functional form used for resource depletion (in (1)) and replenishment (in (3)), as is confirmed by considering the limit of large pathogen loads, where 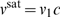; the resulting peak height, 
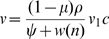
, shows the same monotonic dependence on 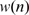 as (5).

Finally, we examine what would happen if cross-immunity or resource limitation were not implemented in the model. Without cross-immunity, 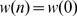, and the initial peak 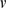 is thus independent of the strain number 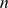 (cf. Fig. S1-2I in Text S1, Sect. B.1). Without resource limitation, (4) degenerates, and the initial peak load cannot be compared for different values of antigenic variation.

### Between-host model

As discussed in the text, we use the basic reproductive number 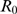 of infected hosts as the measure of evolutionary fitness for infectious diseases [23]. For infections of finite duration 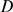,
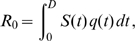
(6)where 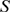 denotes the number of susceptible hosts in the neighborhood (of assumingly constant size 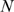) of one initially infected host, and 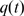 is the transmission rate from the index case at time 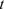 after infection. The pathogen-load dependence of the transmission rate is modeled by
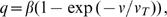
(7)where 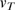 is the infectiousness threshold parameter and 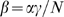 is the transmission coefficient, which critically depends on the contact rate 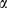. The parameter 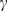 is the transmission probability per contact for a completely saturated pathogen load (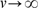), and lies between 0 and 1. This functional form is consistent with data for HIV (Fig. 1). The transmission dynamics in the entire susceptible contact neighborhood of an index case are given by
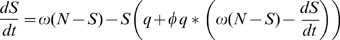
(8)where 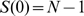. This equation models a local dynamic network (derived in the section below), where 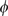 defines the transitivity or cliquishness of the network (proportion of neighbors of a node who are neighbors of each other) and 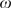 the per-capita rate at which hosts in the neighborhood of the index case are replaced by new susceptible hosts. Here 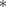 represents convolution, with 
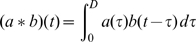
. This expression corrects for the depletion of the local contact neighborhood of the primary case by individuals infected by the index case then infecting shared contacts of the index before the index case herself does. Such local saturation of the susceptible population is entirely a network effect and vanishes for 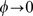.

It should be noted that the network dynamics are invariant for 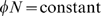, bar a scaling of 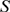 by 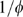. Enlarging the neighborhood size thus corresponds to effectively reducing cliquishness. This relation allows for incorporating vector-borne infections (characterized by large 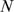) into our classification (as type B infections; cf. end of the section Infection types). Although our modeling framework has been designed for direct transmissions, it can formally be applied to vector-borne infections assuming that (due to relatively low 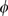) the transmission delay through the vector is less important.

### Network model

Here we derive Eqn. (8) of our between-host model, which also illustrates how the two parameters, 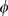 and 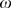, characterize the host-contact network on local and on global scales, respectively.

The transmission dynamic in an initially entire susceptible contact neighborhood of one index case and fixed size, 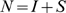, can be reconstructed approximately in terms of average numbers of infectives and susceptibles (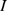 and 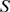, resp.),
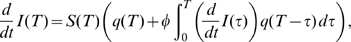
(9)counting the (infinitesimal) number of new infections caused by the index case at time 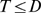. We have included direct infections and secondary infections which, we assume, occur with likelihood 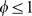 in the contact neighborhood. The time delays, 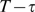, as reflected by the transmission rates relevant for secondary infections, correspond to primary infections at 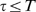. The integral covers the secondary infections caused by new infectives up to time 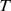, respecting the changing transmission rates resulting from time-dependent pathogen loads (cf. (7)).

Written exclusively in terms of susceptibles (while utilizing the notion of convolution), (9) reads
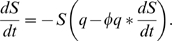
(10)From here, Eqn. (8) is obtained by incorporating a constant (global) flow of individuals (referring to the entire host population) into the transmission model, quantified by the replacement rate 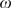 of individuals in the considered neighborhood. This is readily confirmed by the formal replacement (of the ordinary derivative by a covariant version),
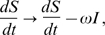
(11)which models the recruitment of new susceptibles in exchange for old infectives.[Table pcbi-1000536-t001]

**Table 1 pcbi-1000536-t001:** Table of parameters.

Parameter	Description
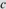	resource
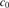	initial/max resource
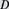	duration of infection
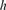	Hill function, 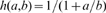
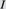	infectives
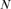	neighborhood size
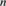	number of mutant strains
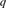	transmission rate
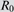	reproduction number
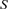	susceptibles
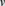	total pathogen load
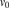	initial/min pathogen load
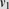	pathogen units per resource unit
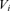	load of strain 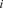
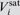	saturated growth of pathogen, 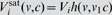
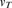	infectiousness threshold (transmission space)
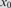	initial/min immunity
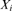	specific immunity to strain 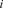
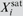	saturated growth of immunity, 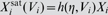
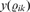	cross-weight (over antigenic distance between strains 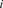 and 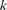 )
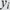	cross-reactive immunity to strain 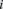 , 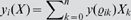
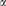	contact rate (transmission space)
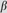	transmission coefficient
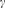	probability of transmission
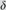	antigenic variation (pathogen space)
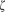	growth of immunity
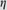	critical load for saturated immune response
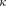	replenishment of resource
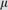	mutation rate
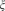	decline of immunity
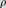	replication rate (pathogen space)
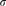	clearance rate of pathogen induced by immunity
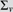	cumulative pathogen load
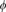	cliquishness (contact space)
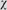	degree of cross-immunity
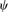	clearance rate of pathogen
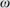	replacement rate (contact space)

## Supporting Information

Text S1 In this appendix we present an extension of our within-host model regarding the implementation of cross-immunity. We include the acquisition of immunity from antigenically similar strains and re-calculate Fig. 2 and relevant parts of Fig. 3. We obtain very similar results compared to our original model by only adjusting the strength of cross-immunity with respect to the antigenic distance while keeping all other model parameters unchanged. This demonstrates the robustness of our original formulation, where cross-immunity is implemented in a more simplified way. In the total absence of tradeoffs between cross-immunity and peak pathogen load, we show that ChD-like infections are excluded. We also illustrate the relation between infectiousness and within-host replication for type C infections. Supplementary to Fig. 3, we plot the cumulative strain number over pathogen space. This allows us to identify infection-type B as the only candidate where, due to our model limitations, exhaustion of strains might impose an implicit artifactual limit on the duration of an infection.
(0.41 MB PDF)
