# Supplementary material for: Antigenic Diversity, Transmission Mechanisms, and the Evolution of Pathogens
Source: PLoS Comput Biol. 2009 Oct 16;5(10):e1000536. doi: 10.1371/journal.pcbi.1000536 (PMC2759524; doi:10.1371/journal.pcbi.1000536)
Supplement: Text S1 — In this appendix we present an extension of our within-host model regarding the implementation of cross-immunity. We include the acquisition of immunity from antigenically similar strains and re-calculate Fig. 2 and relevant parts of Fig. 3. We obtain very similar results compared to our original model by only adjusting the strength of cross-immunity with respect to the antigenic distance while keeping all other model parameters unchanged. This demonstrates the robustness of our original formulation, where cross-immunity is implemented in a more simplified way. In the total absence of tradeoffs between cross-immunity and peak pathogen load, we show that ChD-like infections are excluded. We also illustrate the relation between infectiousness and within-host replication for type C infections. Supplementary to Fig. 3, we plot the cumulative strain number over pathogen space. This allows us to identify infection-type B as the only candidate where, due to our model limitations, exhaustion of strains might impose an implicit artifactual limit on the duration of an infection. (0.41 MB PDF) [file pcbi.1000536.s001.pdf]

Text S1

Antigenic diversity, transmission mechanisms and the evolution of pathogens

Alexander Lange [1,2,*], **Neil M. Ferguson** [1]

1 MRC Centre for Outbreak Analysis and Modelling, Dept. of Infectious Disease Epidemiology, Imperial College London, UK

2 Dept. of Mathematics & Statistics, McMaster University, Canada

∗ E-mail: alange.publ@gmail.com

Abstract In this appendix we present an extension of our within-host model regarding the implementation of cross-immunity. We include the acquisition of immunity from antigenically similar strains and re-calculate Fig. 2 and relevant parts of Fig. 3.[†] We obtain very similar results compared to our original model by only adjusting the strength of cross-immunity with respect to the antigenic distance while keeping all other model parameters unchanged. This demonstrates the robustness of our original formulation, where cross-immunity is implemented in a more simplified way. In the total absence of tradeoffs between cross-immunity and peak pathogen load, we show that ChD-like infections are excluded. We also illustrate the relation between infectiousness and within-host replication for type C infections. Supplementary to Fig. 3, we plot the cumulative strain number over pathogen space. This allows us to identify infection-type B as the only candidate where, due to our model limitations, exhaustion of strains might impose an implicit artifactual limit on the duration of an infection.

A Extended within-host model

We extend our original within-host model by including the acquisition of immunity from antigenically similar strains. This is done by modifying Eqn. (2), where we incorporate cross-weights $z(\varrho)$ over the involved antigenic distances ϱ, defined analogously to $y(\varrho)$. All the other equations and parameters are kept unchanged.

A.1 Definition

For the pathogen strain $i \geq 0$, the new version of (2) reads

$$\frac{dX_i}{dt} = \xi(x_0 - X_i) + \zeta z_i \circ X_i^{\text{sat}}(V)\,, \tag{S1-1}$$

where $z_i \circ X_i^{\text{sat}}(V) = \sum_{k \leq n} z(\varrho_{ik}) h(\eta, V_k) X_i$ models the acquisition of immunity depending on the pathogen load vector $V = (V_k)_{k \leq n}$, weighted according to the corresponding antigenic distances $z(\varrho_{ik})$. Saturation is taken account of, again through the Hill function $h(a,b) = 1/(1 + a/b)$, and for each strain $k \leq n$ separately.

[†] References to equations and figures in this text are always marked by the hyphenated prefix "S1" — labels without this prefix refer to objects in the main text.

The inclusion of immune acquisition, incorporated into the extended model via the cross-weight function z, leads to an increased presence of immunity. This requires us to reduce the immune reaction by adjusting the cross-weight function y. In numerical simulations (cf. Figs. S1-1–S1-4), we let

$$z(\varrho) = y(\varrho) = \begin{cases} \frac{1}{4} y^{\mathrm{orig}}(\varrho) & \varrho > 0 \\ y^{\mathrm{orig}}(0) & \varrho = 0 \end{cases} \tag{S1-2}$$

where y^{orig} denotes the cross-weight function of our original model in the text. There is no particular requirement to utilize a symmetric-looking definition here; other forms produce similar outcomes. Except for y, all quantities are assumed to be as in the original model.

A.2 Results

The individual and average curves of the extended within-host dynamical model are shown in Fig. S1-1. The subfigures correspond to the ones of the original model in Fig. 2 — each of the 6 pairs capturing obvious similarities. One also confirms that the three characteristic surfaces Figs. 3A,B,C (copied to Figs. S1-2D,E,F) of the original model are similar to the corresponding surfaces of the extended model (Figs. S1-2A,B,C) — both in shape and with respect to the locations of the maxima.

As expected from the original model, type A has the highest pathogen load and shows widely varying individual durations (Figs. S1-1A,B) — leading to characteristic average load curves of very small tails and moderate average durations (Fig. S1-1C). Regions nearby the load maximum in pathogen space lead to shorter individual durations (Figs. S1-2A,B), possibly important for real-world realizations of this type — such as influenza. However, type A is more universal than only representing flu — for infectiousness thresholds above the critical value, $v_T > v_T^{\mathrm{crit}}$, this type is expected to emerge at any contact rate (cf. Figs. 4 and 5). All these results have also been obtained with our original within-host model — the only difference is the location of the load maxima in pathogen parameter space. The maxima of the extended model are shifted towards lower antigenic variation (of about one or two orders of magnitude, cf. Figs. S1-2A,D).

Also for the two other infection types, B and C, the within-host surfaces of the extended model are very similar to the ones of the original model. Again, only the scale of the antigenic variation changes a bit — for both types one observes shifts from intermediate towards lower values (cf. Figs. S1-2B,E and C,F, resp.).

B Tradeoff between initial peak load and antigenic variation

The within-host dynamics imply a tradeoff between initial peak load and antigenic diversity. The relation was discussed and visualized for the original model (Fig. S1-2F), but it is easily recognized for the extended model (Fig. S1-2C). The necessary requirement, as argued in the Methods section, is the implementation of cross-immunity.

B.1 Cross-immunity — requirement for the emergence of ChDs

The characteristic within-host surfaces without cross-immunity are shown in Figs. S1-2G,H,I. Besides huge load values (type A) and extended durations (type B), one observes a flat initial peak surface with respect to the diversity parameter δ. That means that there is no preferred value of antigenic variation and, in particular, diseases with low variation are not favored (selected through the highest R_0 value) by evolution. In other words, without the possibility of cross-immunity ChD-like type C infections are not expected to emerge.

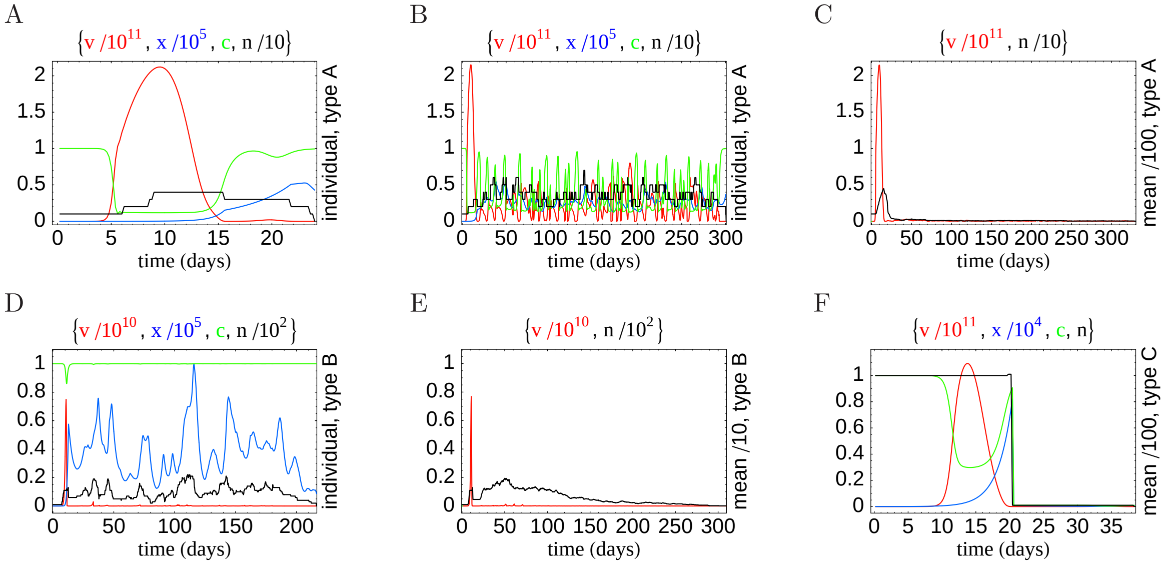

Figure S1-1. Within-host dynamics of the extended model. Graphs show pathogen load [red], specific immunity [blue], resource [green], number of strains [black] and corresponding mean values plotted over time — for individual hosts in (A,B,D) and average hosts in (C,E,F), respectively. (A) and (B) show two different model realizations for the same parameters of antigenic mutation proportion $\delta = 10^{-8}$ and replication rate $\rho = 8$/day, defining type A infections of the extended model, cf. Fig. S1-2A. Similar to the original model, one observes extremely different durations of infection — reaching from a few days up to one year. (C) shows the corresponding average behavior over 100 realizations, characterized by low pathogen loads at large times. This infection type thus corresponds to intermediate and low mean durations of infection — much shorter than the approached maximum of one year. This is also reflected by the mean strain number, which reaches a maximum of 5 at the initial peak, drops down to very low values (< 1) where it stays for several months. (D) and (E) show the pathogen dynamics specific for type B infections with $\delta = 10^{-3}$ and $\rho = 3$/day, for individual and average hosts, respectively. The mean values of load and strain number coincide with the individual values, which confirms long durations and large strain numbers as characteristic trait of this infection type. (F) illustrates type C infections through average curves (over 100 runs) at $\delta = 10^{-9}$ and $\rho = 3$/day; mean and individual values coincide almost identically for the average duration (of ca. 20 days) where the average strain number is close to 1. Longer durations (up to ca. 40 days) are possible for a very small number of cases.

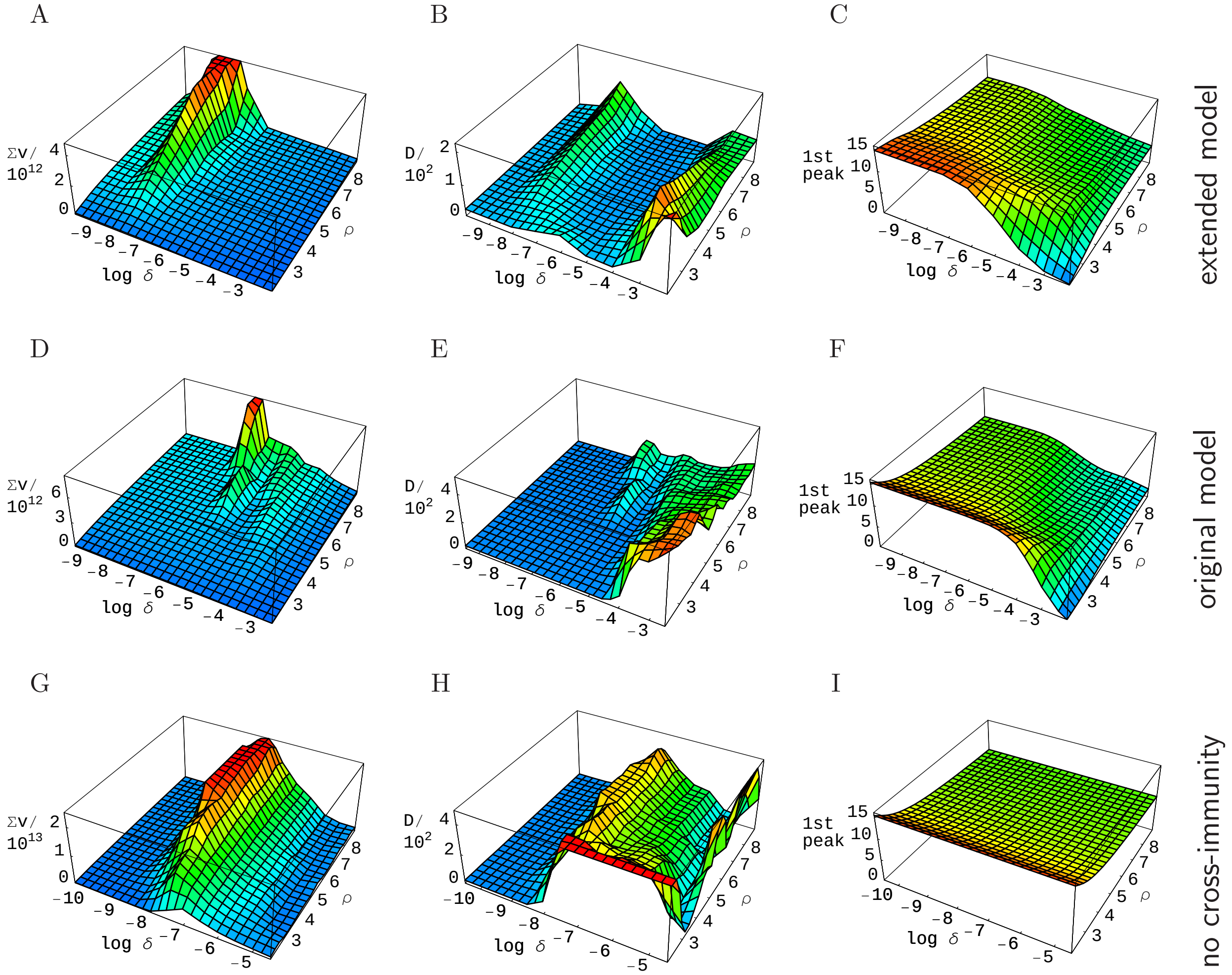

Figure S1- 2. Within-host characteristics for different implementations of cross-immunity. Similar to Fig. 3, the three columns show the cumulative pathogen load (A,D,G), the duration of infection (B,E,H), and the combination of the latter two at the initial peak (C,F,I) plotted over pathogen parameter space, $(\log\delta, \rho)$; in fact, the Figs. S1-2D,E,F are copies of the Figs. 3A,B,C, resp., representing the original model. The results of the extended model are shown in the upper row (A,B,C); the shapes of the surfaces are similar to the original model (despite shifts, $|\Delta \log \delta| \lesssim 2$, $|\Delta\rho| \lesssim 2$) and also the locations of the maxima coincide, corresponding to our infection-type classification. Only the bottom row, which shows the characteristic surfaces without implementation of cross-immunity (i.e., $z(\varrho_{ik}) = y(\varrho_{ik}) = \delta_{ik}$), is different from the upper two rows — in particular, Fig. S1-2I. The surface in (I) is flat with respect to the parameter δ — ChD-like type C infections cannot be identified (at low antigenic diversity).

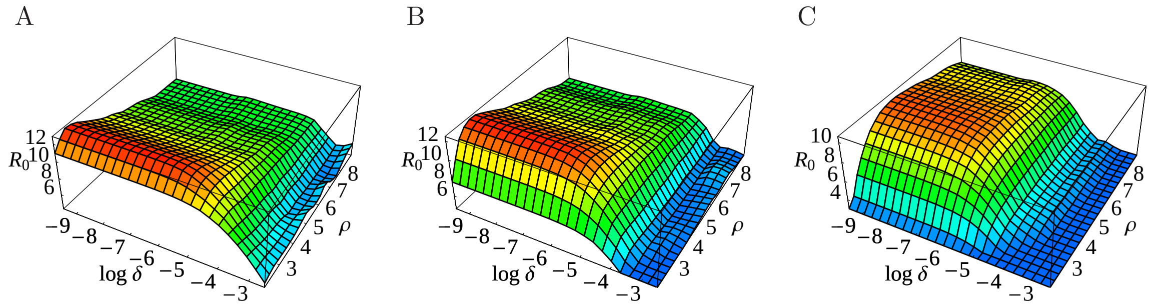

Figure S1-3. Relation between infectiousness and within-host replication. Between-host dynamics, R_0, for different infectiousness values: (A) $v_T = 10^9$, (B) $v_T = 10^{10}$, (C) $v_T = 10^{11}$, illustrated for type C infections (i.e., contact rate $\alpha = 1$) with parameter values as in Fig. 3. Within-host replication (represented by maximal R_0) is monotonically increasing with respect to infectiousness: (A) $\rho = 3$, (B) $\rho = 3.5$, (C) $\rho = 5$.

B.2 Relation between infectiousness and within-host replication

Infectiousness v_T and the favored within-host replication rate ρ are directly correlated. This is obvious when comparing type A and B infections — the former showing high rates ρ (> 8) while representing the limit $v_T \to \infty$ and the latter showing low rates ρ (< 3) while representing the limit $v_T \to 0$. The statement is less obvious when including type C infections, which occur at high infectiousness values but show rather low replication rates (in Figs. 3F and 5D). The apparent contradiction is resolved by noticing that type C infections occur within the v_T-range of type A. In Fig. S1-3, we calculate Fig. 3F again for various infectiousness values $v_T > 10^8$, which also leads to replication rates $\rho > 3$. This confirms our statement also within type C infections.

C Robustness of the type classification

After altering the implementation of cross-immunity, we also demonstrate that limitations concerning the way we model antigenic variation are not important for our infection-type classification result.

The choice of 5 loci with 3 alleles was imposed by the computational complications of solving models with larger numbers of strains. This choice allows for a maximum of 243 different antigenic strains to be generated by random mutations, which — even if back-mutations are possible — is relatively small for high mutation rates. The cumulative numbers of strains generated during an infection are shown in Fig. S1-4; one observes saturation effects for high mutation rates (which characterize type B), especially for high replication rates (cf. Figs. S1-4C,D). Exhaustion of strains resulting in an early end of infection is therefore a potential limitation of our model — possibly affecting type B infections. However, such truncation of the duration has not been seen for this type of infections, which are also characterized by long durations even with limited number of strains modeled. Therefore, even if model limitations are artificially truncating the duration of those infections, this is not affecting the overall self-organization of pathogen types generated by the model. The types A and C are characterized by low average numbers of strains, which means, these types are unaffected by limits on strain numbers.

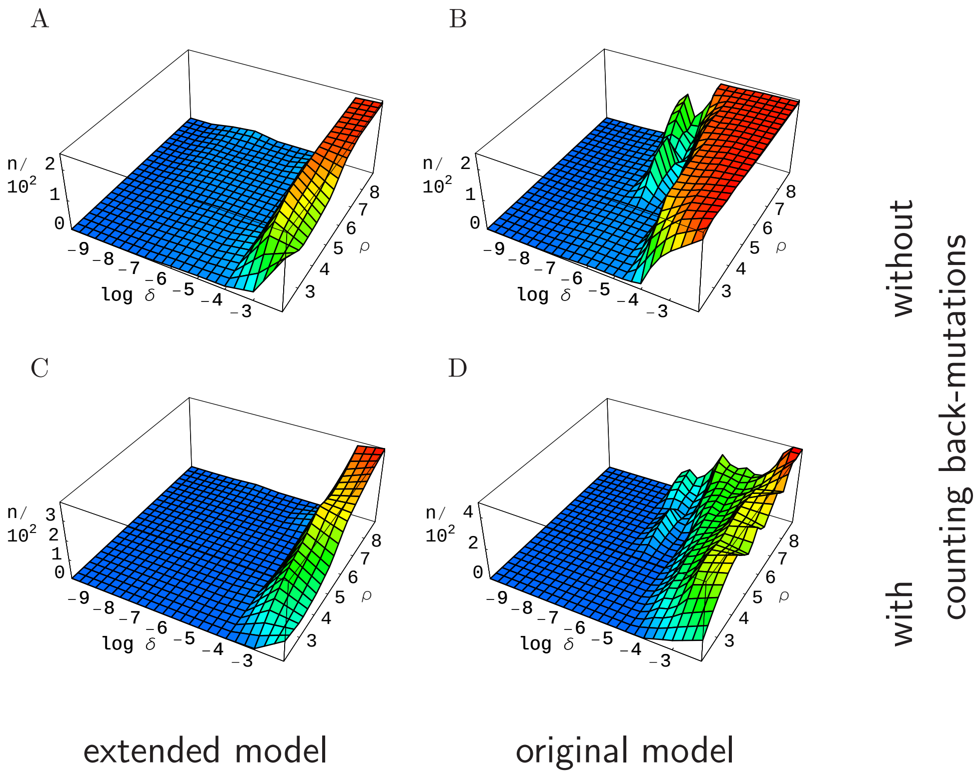

Figure S1-4. Cumulative strain numbers over pathogen parameter space. For both the original (B,D) and extended (A,C) model strain numbers are shown with (C,D) and without counting (A,B) back mutations. Saturation effects are seen for high antigenic variation (A,B); however, this may only lead to exhaustion and an early end of disease at high replication rates (C,D).
